# A survey of coping strategies and resilience in women victims of domestic violence during the COVID‐19 pandemic in Tehran, 2020

**DOI:** 10.1002/brb3.2730

**Published:** 2022-08-02

**Authors:** Farzaneh Rashidi Fakari, Mahbobeh Ahmadi Doulabi, Tahereh Mokhtaryan‐Gilani, Alireza Akbarzadeh Baghban, Sepideh Hajian

**Affiliations:** ^1^ Department of Midwifery, School of Medicine North Khorasan University of Medical Sciences Bojnurd Iran; ^2^ Department of Midwifery and Reproductive Health, School of Nursing and Midwifery, Midwifery and Reproductive Health Research Center Shahid Beheshti University of Medical Sciences Tehran Iran; ^3^ Department of Midwifery and Reproductive Health, School of Nursing and Midwifery Shahid Beheshti University of Medical Sciences Tehran Iran; ^4^ Proteomics Research Center, Department of Biostatistics, School of Allied Medical Sciences Shahid Beheshti University of Medical Sciences Tehran Iran

**Keywords:** COVID‐19, domestic violence, quarantine, women

## Abstract

**Background:**

Domestic violence against women is a hidden and global epidemic that has many negative effects. The COVID‐19 pandemic, quarantine, and staying at home can lead to violent and domestic violence against women. Due to the importance of the subject, the present study was conducted to investigate the coping strategies and resilience of women victims of domestic violence in the COVID‐19 epidemic in Tehran, 2020.

**Methods:**

The present study is a descriptive‐analytical study on 420 women who suffered domestic violence in Tehran, 2020. Data collection tools included a demographic information form, socioeconomic status questionnaire, WHO standardized domestic violence questionnaire, Connor–Davidson Resilience Scale, and Endler and Parker's coping strategies questionnaire. This study was based on the Internet and online. The research poster, the characteristics of the participants, the voluntary participation in the study, the confidentiality of the information, and the link to the online questionnaire were made public through Internet networks.

**Results:**

There was no correlation between resilience with general violence (*p =*.221), types of physical violence (*p =*.502), psychological violence (*p =*.178), and sexual violence (*p =*.386). The results also showed that there was a statistically significant difference between the women who were using or not using a problem‐oriented style with physical violence, using or not using an emotion‐oriented style with physical, psychological, sexual violence, and using or not using an avoidance style with physical violence among the samples (*p* < .05).

**Conclusion:**

The use of coping strategies leads to a reduction in domestic violence against women during the COVID‐19 pandemic. Therefore, designing and implementing training programs to improve coping styles in women can be effective in dealing with such stressful situations and help prevent injuries caused by violence.

## Graphical abstract

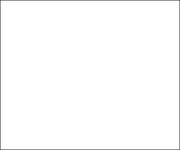

## 1 INTRODUCTION

Domestic violence against women is a concealed, chronic, and comprehensive epidemic in all human societies and has many disadvantages (Rakovec‐Felser, 2014; Rivera et al., 2015). Domestic violence is savage behavior that causes hurt or is accompanied by the probability of physical, sexual, and psychological harm, as well as economic pressure imposed by an adult on a person with whom he or she has a close relationship (Ghazanfarpour et al., 2021; Pence, 1983; Rivera et al., 2015; World Health Organization, 2013). Violence against women can result in physical injuries such as all types of bruisings, fractures, psychological hurts such as depression, anxiety, and posttraumatic stress disorder, obsessive‐compulsive disorder, and suicidal behaviors, and it can also reduce resilience, anxiety tolerance, and stress in women (Pence, 1983). It is estimated that throughout the world, approximately 30% of women experience domestic violence (Devries et al., 2013). On average, in a systematic study in Iran, the prevalence of this problem was reported to be 22.9%, and Tehran had the highest prevalence of domestic violence among other cities (Adineh et al., 2016). There are reports about an increase in domestic violence following COVID‐19. (Campbell, 2020; Telles et al., 2020). The COVID‐19 pandemic and the ‘‘stay at home’’ instruction can affect all of these items and prepare the environment for domestic violence against women (Poate, 2020). Around the world, it is reported after social isolation and quarantine measures, and there has been intimate partner violence against women in Australia, the United States, India, and Brazil (Boserup et al., 2020; Malathesh et al., 2020; Marques et al., 2020; Poate, 2020).

Sufficient and inclusive interventions for women who suffer from domestic violence require a multiaspect attitude, and the combination of interventions is based on the needs of women who undergo violence, care providers, and social factors (Asadi‐Bidmeshki et al., 2020; Kiani et al., 2021). Women who are domestic violence victims usually apply emotion‐oriented strategies to cope with domestic violence or its outcomes, and they mostly do not have problem‐solving strategies (Waldrop & Resick, 2004; World Health Organization, 2013). Two main approaches to deal with stress are defined, which are known as problem‐solving‐oriented and emotion‐oriented methods. In a problem‐solving‐oriented coping strategy, the person focuses on the problem and endeavors to solve it. Conversely, the emotion‐oriented method explains a way in which an individual concentrates on himself/herself, and all his/her struggles are focused on decreasing his/her emotions (Lazarus, 2013). In some sources, the third method is presented as avoidance style, which is distracting thoughts and amusing or trying to absorb in a new activity and entertaining by doing some social activities (Krok, 2015).

Positive adaptation in response to unpleasant conditions (Fleming & Ledogar, 2008; Ungar & Perry, 2012) is resilience. Resilience means the ability of a person to adapt or overcome disasters and pressures, even being reinforced by those experiences. This character is strengthened by a person's inner apt, social skills, his/her interaction with the environment, and his/her spirituality, and it is noticeable as a positive trait (Diener & Suh, 2000; Pietrzak & Southwick, 2011; Zautra et al., 2010).

In the current pandemic and social isolation, women who are victims of domestic violence cannot apply approaches such as running away from the situation and getting support from health systems (Marques et al., 2020; Waldrop & Resick, 2004). The stress resulting from the COVID‐19 pandemic and the emphasis on not leaving home prepare the conditions for increasing the possibility of domestic violence against women and can influence the rate and type of women's resilience against domestic violence (Beland et al., 2020; Plomecka et al., 2020).

The coronavirus crisis in the world, staying at home and social distancing enforcement, financial and economic problems, and sedentary life are effective factors that expand anxiety and stress that can result in furious behaviors.

Regarding the importance of the subject, the present study was carried out to investigate the coping strategies style and resilience rate of women who are victims of domestic violence in the COVID‐19 epidemic in Tehran in 2020.

The hypotheses of this study are as follows:

1. Coping styles are associated with all kinds of violence.
2. The rate of resilience is associated with all kinds of violence.

The inclusion criteria comprise one or more types of physical, sexual, and psychological domestic violence in a recent year; married; Iranian race; do not apply opium or psychedelic drugs; no history of the psychological, chronic, and incurable disorder which is diagnosed according to individual statements; and no history of calamity exception of domestic violence in the last 6 months (such as the death of a close relative, family's severe quarrels, financial problems). The exclusion criteria comprise unwillingness to respond to the questionnaires at the time of the study and incomplete fill‐in of the questionnaires.

## 2 MATERIALS AND METHODS

The present study is a descriptive‐analytical study based on Internet network. Sampling was based on purpose and online using official social and popular networks. The final sample was calculated 420 people (α = 0.05, *z*_α/2_ = 1.96, δ = 9.308, *d* = 1), According to the study of Hajian et al. (2018).

The research questionnaires comprised the questionnaire of demographic and socioeconomic characteristics, the questionnaire of the socioeconomic status of Ghodratnama, the WHO standardized study of domestic violence questionnaire, the Connor–Davidson Resilience Scale, and the coping strategies questionnaire of Endler and Parker.

The questionnaire of demographic and socioeconomic characteristics includes age, age of marriage, history of a previous marriage in each couple, age of spouse, level of education, occupation, monthly income of the family, housing status, space of the house, and the number of family members considered in the COVID‐19 pandemic. Its validity was determined by the qualitative content validity method; it was given to six faculty members of the Department of Midwifery and Reproductive Health and six faculty members of Psychiatry and Psychology of Shahid Beheshti University of Medical Sciences. Some questions were refined.

The questionnaire of the socioeconomic status of Ghodratnama has four parts: income rate, economic level, education, housing status; six demographic questions; and five main questions. The validity of the questionnaire was proven by Eslami et al. (2014). Using Cronbach's alpha test, the reliability of the questionnaire was 0.83 (Eslami et al., 2014).

The WHO standardized study of domestic violence questionnaire includes the dimensions of physical (nine items), sexual (five items), and psychological‐emotional violence (11 items) to consider the type and severity of intimate partner violence as well as to investigate the adopted strategies to cope with/fight violence (nine items) (Garcia‐Moreno et al., 2006). Hajian et al. (2014) verified the validity of the questionnaire. Additionally, internal consistency was acquired using Cronbach's alpha coefficient for physical, sexual, and psychological violence, and the adopted strategies to cope with/fight violence were 0.92, 0.81, 0.89, and 0.88, respectively (Hajian et al., 2014).

*Connor–Davidson Resilience Scale*: This scale has 25 components and five subscales representing personal competence/personal tenacity, trust in one's instincts/tolerance to negative affect, positive acceptance of change/secure relationships with others, control, and spiritual influences. Responses are scored on a Likert scale from 0 (never) to 4 (always). The minimum score is zero, and the maximum is 100 (Connor & Davidson, 2003). Samani et al. (2007) proved the validity of this questionnaire. The reliability of this was verified by internal consistency and Cronbach's alpha coefficient (0.93).

The coping strategies questionnaire of Endler and Parker has 48 items and assesses stress coping strategies in three dimensions: problem‐solving (16 items), emotion‐oriented (16 items), and avoidance (16 items). Individual responses were scored on a Likert scale from 1 (never) to 5 (always) (Endler & Parker, 1994). The range of scores for each coping strategy varies from 16 to 80. By the same token, regarding the highest scores obtained from the questionnaire, the individual's dominant coping style is determined, and each behavior that acquires a higher score is considered the individual's coping style with stress.

Ghoreyshi Rad (2010) confirmed the validity of this questionnaire. Additionally, by the internal consistency and calculating Cronbach's alpha coefficient (0.83 for the whole tool and 0.86, 0.81, and 0.79 for subscales: problem‐solving, emotion‐oriented, and avoidance, respectively), the reliability of this tool was proven by Ghoreyshi Rad (2010).

Information was collected after approving the proposal and obtaining allowance from the ethics committee of Shahid Beheshti University of Medical Sciences and taking the necessary permits from the University of Medical Sciences and the School of Nursing and Midwifery of Shahid Beheshti. In conclusion, this study was carried out using Internet network‐based and official and popular online social media. Questionnaires were designed on the Google platform. The research poster that included a brief introduction to the research background, research objectives, characteristics of participants, voluntary participation in the study, the confidentiality of the information, and the link to the online questionnaire was announced by social networks. At the beginning of the questionnaires, if participants were willing and eligible, they signed the informed consent form to take part in the study. Women who did not report any violence were pondered as those who did not experience intimate partner violence and were not considered at all. If the participant experienced at least one type of violence in each of the areas (physical, psychological, sexual) during the COVID‐19 period and the recent 1 year since the study, relying on the type of violence was included in the study. If a woman experienced each type of violence during the COVID‐19 period (over the past year) since the study, at least one to two times: mild violence, three to five times: moderate violence, and more than five times: severe violence was classified.

Statistical analysis was performed using SPSS software version 20. First, the normality of quantitative variables was specified by Kolmogorov–Smirnov and Shapiro‒Wilk tests. Descriptive statistics to prepare tables, calculate percentage, mean, standard deviation, and inferential statistics to analyze and find relationships were applied. The Pearson coefficient was used to examine the correlation between variables, and regression models were used to determine the relationships between independent and dependent variables. The dependent two‐dimensional variable includes physical/psychological/sexual violence (yes–no), and independent variables include coping strategies and resilience, and so on. Thus, to check the relationship between independent and dependent variables as well as to adjust the effect of other variables, this model was used. On the other hand, the purpose of using logistic regression was to support the research hypotheses (significance level of tests was considered less than 0.05).

### 2.1 Ethical approval committee

Consent was taken from all participants to participate in the research. This study is part of a research project approved by Shahid Beheshti University of Medical Sciences with the ethics code IR.SBMU.PHARMACY.REC.1399.130.

## 3 RESULTS

The results showed that the mean age of women victims of domestic violence in COVID‐19 was 36.24 ± 8.6 years, and the mean age of the spouse was 40.07 ± 9 years. Most women were between the ages of 29–34, and their spouses were 33–43. According to the Ghodratnama questionnaire, the socioeconomic status of the majority of women was at the middle level (48.1%).

Education of the majority of women (77.6%, N = 326) and their spouses (66.4%, N = 279) was university, the majority of women's careers (55.5%, N = 233) was housewives, and their husbands (41.9%, N = 176) were self‐employed. In most cases, the economic status was less than sufficient (50.5%, N = 212), the family income was more than three million Tomans per month (74.8%, N = 314), and affordability to buy a house was low (if they do not possess a house) (52.1%, N = 219).

The mean number of pregnancies was 1.77 ± 1.33, delivery was 1.88 ± 3.45, and abortion was 0.48 ± 0.82. A total of 8.8% (N = 37) of women reported a history of infertility. The majority of women (35.7%, N = 150) reported having sex twice a week. A total of 21% (N = 88) of women and 8.1% (N = 34) of their husbands contracted COVID‐19. In the majority of cases, keeping away coitus for punishment (68.3%, N = 287), dissatisfaction of sexual intercourse humiliatingly (84%, N = 353), asking intercourse without consent (61%, N = 256), the use of coercion to have sex (81.2%, N = 341), asking unusual and without agreement intercourse (82.1%, N = 345) never happened. The prevalence of physical violence was 55.7%, psychological violence was 66.7%, and sexual violence was 47.1%.

Table 1 shows the frequency of all violence among women who were victims of domestic violence during the COVID‐19 pandemic (Table 1).

**TABLE 1:** Distribution of absolute and relative frequencies of participants according to the type of violence by their spouse

Variable		Percentage (number)
Physical violence	No violence	186 (44.3)
	Mild	180 (42.9)
	Moderate	53 (12.6)
	Sever	1 (0.2)
	Total	420 (100)
Psychological violence	No violence	140 (33.3)
	Mild	200 (47.6)
	Moderate	54 (12.9)
	Sever	26 (6.2)
	Total	420 (100)
Sexual violence	No violence	222 (52.9)
	Mild	142 (33.8)
	Moderate	43 (10.2)
	Sever	13 (3.1)
	Total	420 (100)

Table 2 shows the frequency of the reason for violence by the spouse, in which the most common was a lack of cultural misunderstanding among couples (38.8%).

**TABLE 2:** Frequency distribution of the cause of violence by the spouse in the participants

Variable	Percentage (number)
Spouse pessimism	96 (22.9)
Lack of cultural understanding with spouse	163 (38.8)
Lack of religious understanding with the spouse	83 (19.8)
Street quarrel	26 (6.2)
Your family interference	43 (10.2)
Spouse family interference	143 (43.0)
Remarriage of spouse	26 (4)
Children from the woman's previous marriage	19 (4.5)
Children from the previous marriage of the spouse	23 (5.5)

The mean and standard deviation of the resilience score and the problem‐solving‐oriented, emotion‐oriented, and avoidance‐oriented styles were 78.16 ± 13.23, 44.65 ± 8.58, 50.89 ± 10.24, and 8 ± 07/46, respectively. Regarding the results of the Pearson correlation coefficient test, between resilience and total violence (*p* = .221), physical (*p* = .502), psychological (*p* = .178), and sexual (*p* = .386) violence, there was no correlation. According to the results of the Pearson correlation test between coping styles with total violence (*r* = −0.052, *p* < .0001), physical (*r* = −0.059, *p* < .0001), and sexual (*r* = 0.071, *r* = 00010) violence, there was a significant inverse correlation. The Pearson correlation between coping styles and all violence is depicted in Table 3.

**TABLE 3:** Correlation between all types of coping styles and all kinds of violence in participants

Variable		Sexual violence	Psychological violence	Physical violence
Yes	Problem‐oriented	44.06 ± 8.98	43.97 ± 8.87	43.62 ± 8.85
No		45.17 ± 8.2	45.50 ± 8.15	46.99 ± 7.66
*p*‐Value and type of test: *t*‐test		0.184	0.069	0.0001
Yes	Emotional‐oriented	48.91 ± 10.87	49.14 ± 10.52	43.62 ± 8.85
No		52.65 ± 9.31	53.09 ± 9.44	55.94 ± 8.62
*p*‐Value and type of test: *t*‐test		0.0001	0.0001	0.0001
Yes	Avoidance‐oriented	46.23 ± 8.85	45.85 ± 8.41	45.21 ± 8.40
No		45.92 ± 7.66	46.38 ± 8.03	47.78 ± 7.64
*p*‐Value and type of test: *t*‐test		0.706	0.492	0.002

The mean physical, psychological, and sexual violence scores in women using problem‐oriented, emotion‐oriented, and avoidance‐oriented styles was lower than women not using these strategies (except avoidance style in sexual violence). The results of the *t*‐test showed a statistically significant difference using or not using a problem‐oriented style with physical violence, using or not using an emotion‐oriented style with physical, psychological, sexual violence, and using or not using an avoidance‐oriented style with physical violence in considered women (05/0 > *p*) (Table 4).

**TABLE 4:** Comparison of the mean score of all types of coping styles with all types of violence in the participants

Variable	Physical violence	Psychological violence	Sexual violence	Total lViolence
Problem‐oriented Style	*r* = −0.590 *p* = .0001	*r* = −0.615 *p* = .025	*r* = −0.071 *p* = .0001	*r* = −0.052 *p* = .0001
Emotional‐oriented Style	*r* = −0.331 *p* = .0001	*r* = −0.352 *p* = .0001	*r* = −0.283 *p* = .0001	*r* = −0.387 *p* = .0001
Avoidance‐oriented Style	*r* = 0.910 *p* = .006	*r* = 0.854 *p* = .009	*r* = 0.417 *p* = −.040	*r* = 0.957 *p* = −.003

The results of the logistic regression test, the total score of resilience, and coping styles with all types of violence are shown in Table 5.

**TABLE 5:** Summary of logistic regression results of the total score of resilience and total score of coping styles with physical, sexual, and psychological violence (yes‐no) in the participants

	Physical violence				Sexual violence				Psychological violence			
Variable	*B*	Beta	Significance	CI	*B*	Beta	Significance	CI	*B*	Beta	Significance	CI
Age of woman	−0.033	0.986	.007	0.945–0.991	0.042	1.043	.104	0.991–1.098	0.042	1.043	.151	0.985–1.106
Age of man	0.009	1.009	.729	0.959–1.061	−0.032	0.986	.563	0.979–1.012	−0.018	0.982	.509	0.903–1.037
Education of woman	0.505	1.65	.047	0.947–0.986	0.395	1.48	.143	0.874–2.521	0.117	1.124	.709	0.608–2.081
Education of woman	0.006	1.069	.786	0.662–1.725	−0.136	0.872	.563	0.550–1.385	−0.079	0.924	.770	0.546–1.565
Problem‐oriented coping style	−0.01	0.990	.505	0.961–1.02	−0.016	0.984	.272	0.956–1.013	−0.01	0.990	.540	0.985–1.023
Emotional‐oriented coping style	−0.035	0.966	.001	0.947–0.986	−0.035	0.966	.002	0.945–0.987	−0.087	0.917	.00	0.895–0940
Avoidance‐oriented coping style	0.01	1.01	.545	0.987–1.042	0.030	1.03	.059	0.999–1.064	−0.015	0.985	.399	0.952–1.020
Resilience	0.004	1.004	.677	0.987–1.02	−0.005	0.995	.563	0.979–1.012	−0.002	0.998	.807	0.979–1.016

^∗^Significant at the 0.05 level (No = 0, Yes = 1; no physical, sexual, psychological violence). Diplomma or Less = 1. Higher than Diplomma = 0. Method = Backward stepwise (Wald).

Emotion‐oriented coping styles were significantly associated with all three types of physical, sexual, and psychological violence (*p* = .001 and *p* = .002, and *p* = .00). Regarding the results, the variables of women's education and age have a significant relationship with physical violence (*p* = .007 and *p* = .0.047). In other words, by increasing the age of women by 1 year, 0.033 units of physical violence decreased. In contrast, women's education (diploma and less) compared to higher education increased physical violence by approximately 0.505 units.

## 4 DISCUSSION

This study investigated the coping strategies and resilience of women who experienced domestic violence during the COVID‐19 pandemic.

In this study, the prevalence of physical violence was 55.7%, psychological violence was 66.7%, and sexual violence was 47.1%. The results of a study in Australia showed that during the COVID‐19 pandemic, approximately 6% of women suffered from coercive control, and 11.6% of women experienced at least one type of psychological or control abuse (Boxall et al., 2020). Another study showed that the prevalence of violence during COVID‐19 was 24.6%, and the highest type of violence was psychological (13.3%) (Gebrewahd et al., 2020).

The results showed that the mean score of all kinds of violence (except the mean score of sexual violence in people with avoidance strategies) in women using coping strategies was lower than that in women not using them. The results of Waldrop and Resick's study showed that women victims of violence used avoidance coping strategies in the encounter of violence (Waldrop & Resick, 2004). The findings of another study showed that women in the face of violence have used avoidance‐oriented coping strategies with a focus on emotions (Pérez‐Tarrés et al., 2017). In selecting coping strategies to deal with violence, various factors and phenomena, such as the source of internal and external control, are effective. If women do not have control over events, they will choose avoidance strategies based on their emotions that preclude them from opting for more impressive solutions to violence (Pérez‐Tarrés et al., 2017).

In this study, there was a statistically significant inverse relationship between problem‐oriented style with physical, sexual and total violence in women. The results of a study in Atlantic coastal states showed that the use of emotion‐oriented strategies was more effective against physical violence (Bauman et al., 2018). Another study stated that the most common problem‐oriented coping styles help the least in coping with violence (Goodman et al., 2003). Additionally, a study demonstrated that women were willing to use emotional and avoidance coping strategies more. Strategies of avoidance and acceptance of responsibility are part of emotion‐oriented coping styles that a person applies to balance emotions and feelings as well as to control emotional reactions (Endler & Parket, 1994). A study showed that the use of problem‐oriented coping strategies was associated with the degree of violence, rigor, harassment, power, and control of the struggle (Sabina & Tindale, 2008). The results of studies stated that women are unaware of the most effective strategies and use impractical strategies (Bauman et al., 2008). Coping is a moderating process that diminishes or removes the negative impacts of stressors (Crockett et al., 2007). Coping strategies are behavioral and cognitive endeavors to meet demands that arise in stressful situations. In the problem‐oriented style, they are actively implemented to solve the arisen problem. Efforts that are fulfilled for an emotional reaction to such needs, and emotion styles rather than problem‐oriented, control emotions (Thoits, 1995). People, by choosing an escape coping style from past events, do not solve problems but temporarily ignore them and avoid encountering and solving them. Finally, they endanger their mental well‐being (Forbush & Watson, 2006).

The results of this study did not show a statistically significant relationship between the mean score of resilience and violence. One of the determinants of people's responses to stressful events such as domestic violence is resilience (Bonanno et al., 2007). Resilient individuals are equipped with a set of common traits that prepare them to conquer the changes and ups and downs of life (Luthar et al., 2000). Resilience is also known as a dynamic process in which psychological, social, environmental, and biological factors interact to enable a person, at any stage of life, to grow, maintain, or recover their mental health even if exposed to hardness (Wathen et al., 2012). The results of a study showed that the rate of resilience in women victims of domestic violence was lower than that in the general population (Tsirigotis & Łuczak, 2018).

The results showed that there was a statistically significant relationship between education and physical violence, which was the same as the results of various studies (Xu et al., 2005; Walton‐Moss et al., 2005). It can be concluded that a low level of education is illustrative that women are not aware of their social rights, and this will result in physical violence by their husbands. Additionally, most women with low levels of education are housewives and less employed, which in turn makes women financially dependent on their husbands and makes them repeatedly abused by their husbands.

The results showed that there was a statistically significant relationship between female age and physical violence, which was the same as the results of different studies (Taft et al., 2015; Van Parys et al., 2014). Young age in mothers results in a lack of experience and skills in problem solving and a lack of intellectual and social maturity, which in itself is a predisposing factor for disclosing violence.

One of the limitations of the present study was that due to COVID‐19 and quarantine, the researchers used electronic questionnaires, in which many women with lower economic levels may not have access to the Internet, leading to bias. Another limitation was the sensitivity of the subject, which could result in bias in the response of individuals. It is suggested that more studies be done in different cultures regarding other important underlying factors.

## 5 CONCLUSION

The application of coping styles by women plays a crucial role in decreasing domestic violence during the COVID‐19 pandemic. Consequently, planning and implementing educational programs to rectify coping styles in women can play an essential role in reducing violence against women in quarantine and pandemic conditions.

## CONFLICT OF INTEREST

The authors declare no conflict of interest.

### PEER REVIEW

The peer review history for this article is available at: https://publons.com/publon/10.1002/brb3.2730.

## Data Availability

The data that support the findings of this study are available on request from the corresponding author.
